# Supplementary material for: Complexome profiling of the Chlamydomonas psb28 mutant reveals TEF5 as an early PSII assembly factor
Source: Plant Cell. 2025 Mar 17;37(6):koaf055. doi: 10.1093/plcell/koaf055 (PMC12164590; doi:10.1093/plcell/koaf055)
Supplement: koaf055_Supplementary_Data [file koaf055_supplementary_data.zip › psb28_interactive.zip!/plots/intensity/qualityPlots/peptides/HSP90A_Cre09.g386750.t1.2.html]

### Cre09.g386750.t1.2

Trivial name: HSP90A  
SeqCoverage [median]: 5.20  
Mass: 80.68 kDa  
  
 

| Sequence | MissedCleavage | Mass [Da] | wtIntensity | muIntensity | Score |
| --- | --- | --- | --- | --- | --- |
| DTEGEPLGR | 0 | 972.45 | 284.86 | 0.00 | 71.50 |
| GIVDSEDLPLNISR | 0 | 1526.79 | 626.46 | 3373.38 | 142.07 |
|  |  |  | 911.3 | 3373.4 |  |
